# COVID-19 and air pollution in Vienna—a time series approach

**DOI:** 10.1007/s00508-021-01881-4

**Published:** 2021-05-06

**Authors:** Hanns Moshammer, Michael Poteser, Hans-Peter Hutter

**Affiliations:** 1 Department of Environmental Health, Center for Public Health, Medical University Vienna, Kinderspitalgasse 15, 1090 Vienna, Austria; 2 Department of Hygiene, Medical University of Karakalpakstan, Uzbekistan, 230100 Nukus, Uzbekistan

**Keywords:** SARS-CoV-2, Infection risk, Nitrogen dioxide, Particulate matter, Acute effects

## Abstract

We performed a time series analysis in Vienna, Austria, investigating the temporal association between daily air pollution (nitrogen dioxide, NO_2_ and particulate matter smaller than 10 µm, PM10) concentration and risk of coronavirus disease 2019 (COVID-19) infection and death. Data covering about 2 months (March–April 2020) were retrieved from public databases. Infection risk was defined as the ratio between infected and infectious. In a separate sensitivity analysis different models were applied to estimate the number of infectious people per day. The impact of air pollution was assessed through a linear regression on the natural logarithm of infection risk. Risk of COVID-19 mortality was estimated by Poisson regression. Both pollutants were positively correlated with the risk of infection with the coefficient for NO_2_ being 0.032 and for PM10 0.014. That association was significant for the irritant gas (*p* = 0.012) but not for particles (*p* = 0.22). Pollutants did not affect COVID-19-related mortality. The study findings might have wider implications on an interaction between air pollution and infectious agents.

## Highlights

- High concentration of nitrogen dioxide (NO_2_) increased the risk of coronavirus disease 2019 (COVID-19) infection on the same day.
- High concentration of particulate matter (PM10) also increased the risk but this effect was not significant.
- Interaction between air pollutants and infectious agents might lead to overadditive effects.
- Our results underline the importance of policies aiming at better air quality and of more stringent limit values.

## Introduction

Adverse health effects of air pollution have been repeatedly shown at concentrations that induce demonstrable effects in the experimental setting, e.g. in volunteers exposed in an exposure chamber [1]. This is particularly true for nitrogen dioxide [2] for which we have also demonstrated short-term effects in previous time series studies in Vienna [3, 4]. One explanation of significant effects of the irritant gas in epidemiological studies in the absence of effects in experimental settings would be through an indirect pathway: nitrogen dioxide at typical environmental concentrations would not damage cells of the respiratory epithelium directly but would still increase their susceptibility to other factors including allergens [5] or infectious agents. The latter might be affected through an up-regulation of receptors [6] or several other mechanisms [7–9].

Such an interaction between air pollution and an infectious agent might not be specific to coronavirus infections but key for any infectious agent transmitted through the air including the novel coronavirus SARS-CoV‑2 [10]. The latter allows studying that interaction as it is a novel virus hitting an immunologically naïve population thereby forestalling confounding influences from past contacts with the virus.

In a previous paper [11] we reported higher relative risks of acquiring coronavirus disease 2019 (COVID-19) and of dying from the disease in people living in Vienna residing in a district with higher air pollution (average concentration of the year 2019 for particulate matter smaller than 10 µm, PM10, and nitrogen dioxide, NO_2_). That analysis was based on COVID-19 cases and deaths reported to the epidemiological documentation system of the Vienna Health Authority up to 21 April 2020. Case reports included the date of diagnosis and/or the date of death as well as the residential district of affected persons. The first COVID-19 case in Vienna was diagnosed on 28 February 2020. The first death of a person infected with SARS-CoV-2 in Vienna occurred on 11 March 2020.

Chronic exposure to air pollution may lead to changes in the respiratory system rendering the organs more susceptible to infection and to severe outcomes. Living in a district with on average higher air pollution levels also makes it more likely to experience acute episodes of high air pollution. Therefore, associations between severity and incidence of COVID-10 cases and air pollution, as observed in our other paper, are not necessarily driven by long-term (last year) exposure but may also be a consequence of short-term exposures in close temporal connection to the infection. To investigate this we performed a time series analysis of daily air pollution concentrations on infection and mortality risks examining the same COVID-19 cases. Information on the date of infection was missing as this time point can generally not be determined conclusively. Therefore, based on knowledge from the affected segment of the population in Austria, it was assumed that each case had been infected on average 5 days before diagnosis. In this study we assumed a shorter delay between first symptoms and testing than estimated in our previous analysis [12] because of typically shorter times to diagnosis in an overall urban setting. Using these assumptions, our time series approach investigated 59 days from 23 February 2020 until 21 April 2020 (the last day with epidemiological data available).

We set out to test two hypotheses: (a) the propagation of the viral disease, measured as the ratio between newly infected persons on day x divided by persons already infectious on the same day, is positively affected by same day air pollution (represented by daily average levels of PM10 and NO_2_). (b) Mortality due to COVID-19 is affected by same or previous day air pollution.

## Material and methods

The PM10 in Vienna is regularly monitored at 13 sampling stations and NO_2_ at 16 stations. Daily mean values at each station are reported online by the Vienna Environmental Protection Agency [13]. Daily mean values from every station were extracted from the daily air quality reports and the average concentrations of all stations were calculated.

The daily number of infections displays a strong positive autocorrelation. Indeed, this autocorrelation is a hallmark of an epidemic spread and renders the statistical analysis of temporal associations with other factors like pollutant concentrations rather difficult. Instead, the number of new cases per number of already infectious cases was estimated hoping that this ratio was more stable and would therefore be easier to link to daily air pollution. That ratio would also be less affected by slowly and gradually changing factors like testing capacity. It was assumed that each COVID-19 case was infectious starting 1 day before diagnosis and remained infectious for another 4 days (6 infectious days in total). In a sensitivity analysis also alternative time spans were used to estimate the number of infectious persons: 3 days before and 4 days after the diagnosis (8 infectious days in total) and 3 days before and 2 days after the diagnosis (6 infectious days in total). In order to achieve a near normal distribution the ratios were converted to fit a natural logarithmic scale.

On March 16th, general measures to fight the viral spread including closure of restaurants, schools, and most shops were introduced and cultural events etc. were cancelled. Therefore, two periods of time (“before” versus “during measures”) could be discerned. The lockdown measures had a pronounced effect on social life and on industry and commerce. This led to a reduction in motorized traffic and thus supposedly [14] also to measurable reductions in traffic-related air pollutants, namely NO_2_. We observed a relatively quick change in the behavior as the measures were announced on Friday and went into force on Monday. During the following observation period no substantial additional measures were implemented and the existing measures remained largely in place.

We quantified the impact of air pollution (PM10 and NO_2_ separately) on the abovementioned (ln) ratios that served as estimates of the reproduction number controlling for time period (before or after introduction of measures) using a linear regression model. Day of the week, same day temperature and temperature averaged over the last 14 days [15] were included (separately and together) as possible confounders. We calculated the daily COVID-19 mortality risk by air pollution in a Poisson regression. All persons with an active infection were assumed to be at risk of dying with a COVID-19 diagnosis. Persons were defined as experiencing an active infection for 10 days beginning at the day of diagnosis. All calculations were performed in STATA 15.1 (Stata Corp, College Station, TX, USA).

## Results

**Fig. 1:**
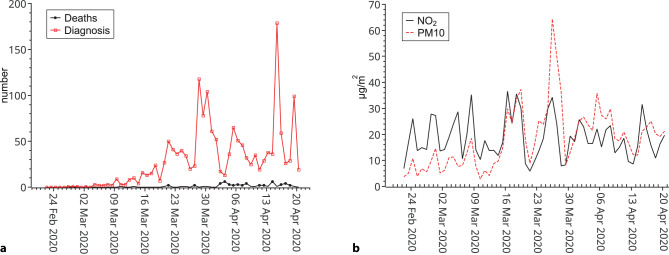
Time course of **a** daily COVID-19 diagnoses and deaths and **b** daily average concentrations of NO_2_ and PM10 in Vienna (23 February 2020 - 21 April 2020)

During 59 days under observation 1665 cases of COVID-19 were diagnosed with a significant increase over time (*p* < 0.001; Fig. 1). On average 28.2 cases occurred per day (range 0–179, SD 33.8). Over the same time 59 deaths of COVID-19 cases occurred: on average 1 per day (range 0–6; SD 1.5). Also, the number of deaths increased significantly over time (*p* < 0.001). The ratio of infected to infectious inhabitants, a rough estimate of the effective reproduction number, was on average 0.36 ranging from 0 to 1.29. It was significantly higher before the measures were in place (0.55 versus 0.26, *p* < 0.001).

Contrary to expectations there was no significant difference in daily NO_2_ levels between the periods before and after the measures were implemented. Therefore, inclusion of the period in the regression model did not substantially change the effect estimates for NO_2_ but increased the precision of the model and thus also of the effect estimate for NO_2_.

**Fig. 2:**
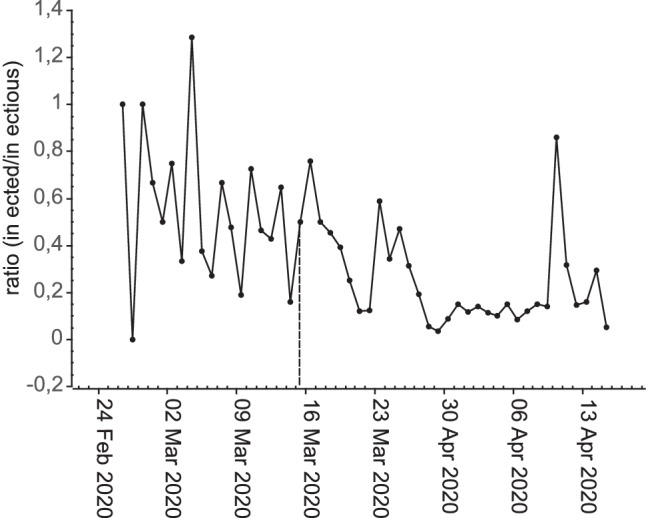
Time course of the estimated ratio of infected by infectious persons in Vienna (23 February 2020 - 21 April 2020) assuming 6 infectious days in total beginning 1 day before diagnosis (March 16th, the onset of measures, marked by a dashed line)

Both NO_2_ and PM10 were significantly (*p* = 0.0001) and positively correlated with each other (R = 0.499). Both pollutants also showed a positive autocorrelation (same day versus preceding day): PM10 *p* < 0.0001, R = 0.716; NO_2_ *p* = 0.021, R = 0.302. The PM10 had an average concentration of 18.2 µg/m^3^ (range 2.9–64.4 µg/m^3^) and NO_2_ of 18.6 µg/m^3^ (range 5.8–36.6 µg/m^3^). The NO_2_ concentrations fluctuated around the same level over the whole observation period while PM10 increased significantly over time from levels below 10 µg/m^3^ in the beginning of the observation period (February 23rd) to levels around 20 µg/m^3^ at the end (April 21st). On March 27th (64.4 µg/m^3^) and 28th (50 µg/m^3^) that trend was interrupted by a peak concentration due to inflow of desert dust from Asia and Africa (personal communication from Austrian Meteorological service; Fig. 1). The ratio infected to infectious declined over time but also displayed considerable fluctuations (Fig. 2).

Same day NO_2_ was significantly and positively associated with the logarithm of the ratio of infected by infectious, while PM10 was not (Table 1). Using alternative estimates of the number of infectious persons per day did not substantially change this finding (Table 2). Also, the inclusion of the day of week and temperature as possible additional confounders did not substantially alter the effect estimates of NO_2_. The effect estimates for NO_2_ and for the period (measures on/off) also remained significant. Also, the effect estimates for PM10 were largely unaffected and remained non-significant.

**Table 1:** Predictors of ln(infected divided by infectious) according to linear regression

Parameter	Coefficient	P‑value	95% Confidence interval
NO_2_ (µg/m^3^)	0.032	0.012	0.007; 0.057
Measures in place	−0.993	< 0.001	−1.411; −0.576
Constant	−1.273	< 0.001	–1.844; −0.702
PM10 (µg/m^3^)	0.014	0.219	−0.008; 0.035
Measures in place	−1.187	< 0.001	−1.744; −0.629
Constant	−0.799	< 0.001	−1.204; −0.394

**Table 2:** Effect estimates using different definitions of “infected”

Definition of “infected”	NO_2_		PM10	
	Coefficient	P‑value	Coefficient	P‑value
1 day before until 4 days after	0.032	0.012	0.014	0.219
3 days before until 4 days after	0.037	0.002	0.011	0.332
3 days before until 2 days after	0.037	0.003	0.011	0.325

Mortality risks from COVID-19 were not affected by PM10 or by NO_2_ (neither same nor previous day concentrations, Table 3).

**Table 3:** Predictors of dying with a diagnosis of COVID-19 (Poisson regression)

Parameter	Coefficient	P‑value
NO_2_ (µg/m^3^) same day	−0.02	0.357
Constant	−5.46	< 0.001
NO_2_ (µg/m^3^) previous day	0.02	0.40
Constant	−6.09	< 0.001
PM10 (µg/m^3^) same day	−0.01	0.456
Constant	−5.54	< 0.001
PM10 (µg/m^3^) previous day	0.01	0.668
Constant	−5.92	< 0.001

## Discussion

Contini and Costabile [16] proposed possible mechanisms linking air pollution with the COVID-19 outbreak risk. They urgently called for robust research studies. Indeed, the body of scientific evidence is growing. Several studies so far investigated the impact of spatial differences in air pollution [17–29] and others have looked into short-term temporal air pollution effects [30–37]. Several possible mechanisms have been proposed that would explain associations between acute and chronic air pollution and COVID-19 risk of infection and of mortality [38–42].

In our other paper [11] we already suggested that an analysis of the temporal association between air pollution and COVID-19 risk would allow pinpointing the actual timing and mechanism of the air pollution effect but we also pointed out the problems of such a time series analysis: the power of such a study would be low because of the short time span under observation and the outcome data, the date of infection would only be known with a large margin of error, most likely inflating the confidence interval of the analysis.

Nevertheless, the success in demonstrating a significant and positive association between spatial variation of air pollution and COVID-19 risk tempted us to try investigating the temporal association as well. The effect estimate for NO_2_ on risk of transmission of infection was robust to the different assumptions regarding duration of infectivity. Furthermore, the size of the effect estimate was plausible. The effect estimates for PM10 were even larger but with a substantially larger confidence interval missing formal significance level thresholds by far. The inability to demonstrate a significant effect of PM10 could be found in the erratic time trend in PM10 concentrations that could not be appropriately controlled for. Also, the peak in concentration caused by desert sand intrusion (a rare event for Vienna) could have confounded any true association: particulate matter from different sources and accordingly with different chemical composition and size distribution could have very different effects on infection risks.

Based on these findings, we expected an effect of air pollution on COVID-19-related mortality. We have demonstrated significant effects of air pollution on mortality before [3, 4] and effect estimates were usually larger the more specific the cause of death. But the more specific the cause of death, the rarer would be the events. This would substantially reduce the power of the data and also render the Poisson model less fitting because of zero inflation. Considering these conditions it is not so surprising that we did not find any clear indication for an air pollution effect on COVID-19 mortality.

Our study only investigated a very short time span. That choice was mostly driven by the previous paper where we investigated the impact of spatial differences in air pollution on COVID-19 infections. We focused on the time period when lockdown measures were in place and the period before. During the lockdown also the mobility within Vienna was much reduced thus increasing the precision of the estimates of personal exposure and district where the infection occurred. The short duration of exposure nevertheless is a problem for a time series analysis because of a reduced study power.

The testing capacity increased moderately and continuously throughout the observation period [43]. This affected both the number of infectious persons and the number of newly infected persons in a similar way. Therefore, we expected no grave impact on the overall effect estimates. Nevertheless, analyses of associations between air pollution and infectious diseases still remain a challenge [44, 45]. This is mainly due to the hallmark characteristic of infectious diseases, namely the spread in clusters that tends to enhance chance events both in space and time and introduces statistical noise. But that noise would more likely obscure a true effect than introduce a spurious one. Another issue is the impact of social distancing that not only affects the virus transmission directly but also might lead to a reduction in air pollution levels [46, 47]. Controlling for the time period before and after the introduction of the nationwide lockdown did improve the model. But lockdown measures did not have a pronounced effect on short-term variation in air pollution levels in Vienna. This is not surprising as also in Munich [48] and in Brescia [49] effects of the lockdown were only seen in roadside stations.

The outcome of such a time series analysis heavily depends on the assumptions regarding the (average) delay between infection and diagnosis. We chose an average delay of 5 days a priori based on limited information from local public health experts. A different choice might have provided different results.

## Conclusion

Evidence for air pollution as an environmental factor increasing the epidemiological adverse impact of COVID-19 is accumulating from studies performed in different parts of the world and under various settings. This study adds to the mounting evidence regarding risk of COVID-19 infection, not of COVID-19-related death. A biological interaction between air pollutants, especially irritant gases like NO_2_ and infectious agents, could further explain epidemiological effects of low concentration air pollutants that usually do not show any effect in experimental studies.
